# Supplementary material for: Association between oral microbiome and seven types of cancers in East Asian population: a two-sample Mendelian randomization analysis
Source: Front Mol Biosci. 2023 Nov 21;10:1327893. doi: 10.3389/fmolb.2023.1327893 (PMC10702768; doi:10.3389/fmolb.2023.1327893)
Supplement: Supplementary file 1 [file DataSheet1.ZIP › Supplementary Materials/MR plots of saliva/saliva═╝/Breast cancer/pheno.1561_to_breast cancer_leave_one_out.pdf]

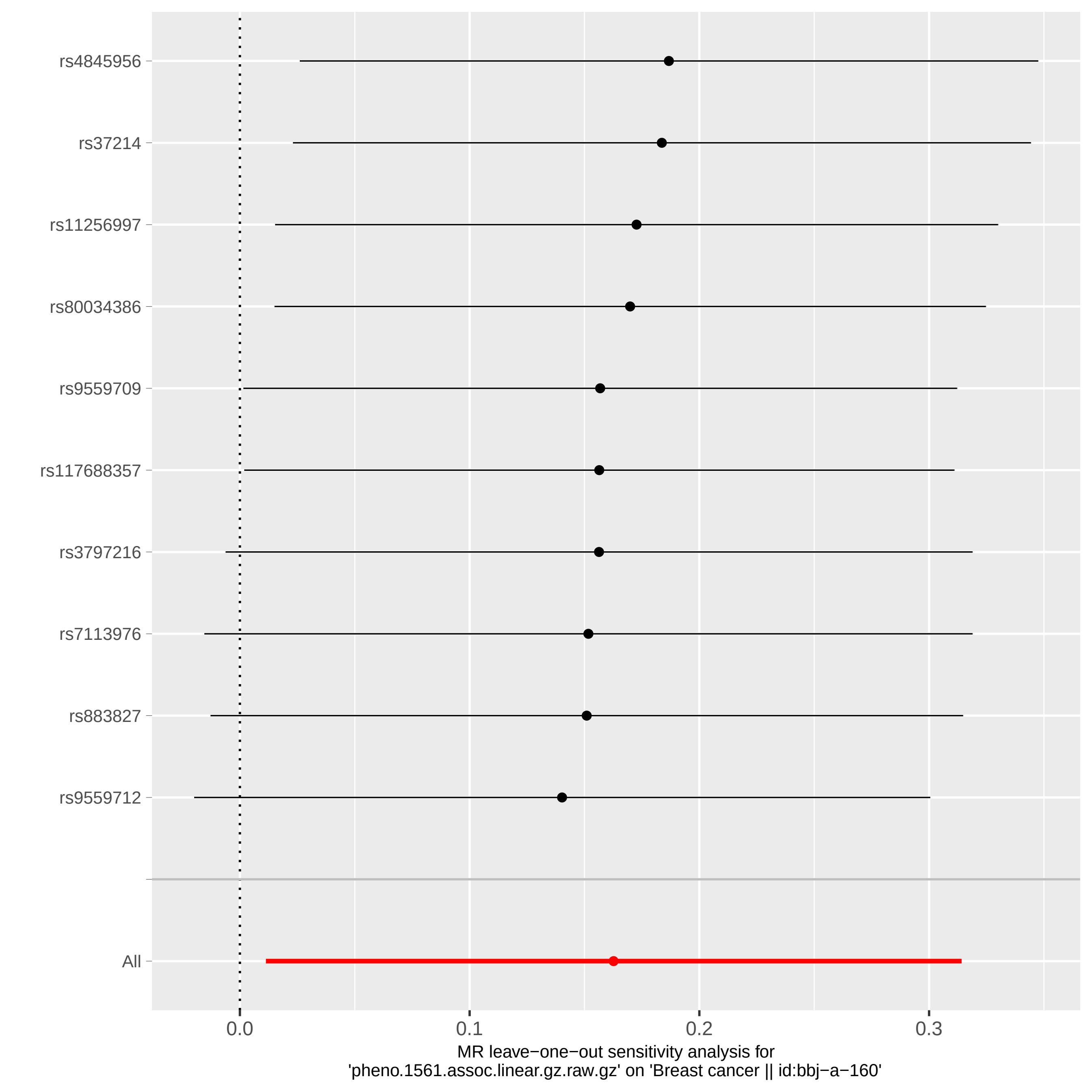

rs4845956
rs37214
rs11256997
rs80034386
rs9559709
rs117688357
rs3797216
rs7113976
rs883827
rs9559712
All
0.0
0.1
0.2
0.3
MR leave−one−out sensitivity analysis for
'pheno.1561.assoc.linear.gz.raw.gz' on 'Breast cancer || id:bbj−a−160'
